# Continuous Percoll Gradient Centrifugation of Erythrocytes—Explanation of Cellular Bands and Compromised Age Separation

**DOI:** 10.3390/cells11081296

**Published:** 2022-04-11

**Authors:** Felix Maurer, Thomas John, Asya Makhro, Anna Bogdanova, Giampaolo Minetti, Christian Wagner, Lars Kaestner

**Affiliations:** 1 Dynamics of Fluids, Experimental Physics, Saarland University, 66123 Saarbrücken, Germany; felixmilan.maurer@uni-saarland.de (F.M.); thomas.john@uni-saarland.de (T.J.); c.wagner@mx.uni-saarland.de (C.W.); 2 Red Blood Cell Research Group, Institute of Veterinary Physiology, University of Zürich, CH-8057 Zürich, Switzerland; asoyersub@gmail.com (A.M.); annab@access.uzh.ch (A.B.); 3 Laboratories of Biochemistry, Department of Biology and Biotechnology “L Spallanzani”, University of Pavia, I-27100 Pavia, Italy; minetti@unipv.it; 4 Physics and Materials Science Research Unit, University of Luxembourg, L-1511 Luxembourg, Luxembourg; 5 Theoretical Medicine and Biosciences, Medical Faculty, Saarland University, 66421 Homburg, Germany

**Keywords:** red blood cells, Percoll, age separation, density gradient, band formation, aggregation, band 4.1 protein, blood sedimentation, complex fluids, micromechanical modeling

## Abstract

(1) Background: When red blood cells are centrifuged in a continuous Percoll-based density gradient, they form discrete bands. While this is a popular approach for red blood cell age separation, the mechanisms involved in banding were unknown. (2) Methods: Percoll centrifugations of red blood cells were performed under various experimental conditions and the resulting distributions analyzed. The age of the red blood cells was measured by determining the protein band 4.1a to 4.1b ratio based on western blots. Red blood cell aggregates, so-called *rouleaux*, were monitored microscopically. A mathematical model for the centrifugation process was developed. (3) Results: The red blood cell band pattern is reproducible but re-centrifugation of sub-bands reveals a new set of bands. This is caused by red blood cell aggregation. Based on the aggregation, our mathematical model predicts the band formation. Suppression of red blood cell aggregation reduces the band formation. (4) Conclusions: The red blood cell band formation in continuous Percoll density gradients could be explained physically by red blood cell aggregate formation. This aggregate formation distorts the density-based red blood cell age separation. Suppressing aggregation by osmotic swelling has a more severe effect on compromising the RBC age separation to a higher degree.

## 1. Introduction

Red blood cell (RBC) sedimentation is a process already observed by the ancient Greeks even before the corpuscular nature of blood in form of cells was known [1]. The sedimentation can be accelerated by applying centrifugal forces. When combined with media of various densities, accelerated sedimentation may be used for cell sorting according to their density. Scientific investigations on these approaches began in the 1960s and it was found that a suspension of silica nanoparticles was the most suited available option [2]. In the following years, improvements were introduced. Among them was the development of modified colloidal silica. Finally, Percoll became a commercial density medium consisting of a suspension of coated silica particles, which were less toxic to cells, non-penetrating, and had a low surface charge [3,4]. Nowadays, Percoll is a standard medium for the density separation of erythrocytes, leukocytes, liver cells, Leydig cells, bone marrow cells, macrophages and other cell types, subcellular particles including plasma membranes and cell organelles, as well as microorganisms such as bacteria, viruses, parasites and algae [5].

As the main corpuscular constituent of human blood RBCs were investigated thoroughly. This includes cell shape [6,7], elastic properties [8], flow properties [9,10], biochemical properties [11,12,13], cellular structure [14], membrane structure [15], ion channels [16,17,18], just to name a few properties. The bending elastic properties of RBCs are a result of the membrane structure [19]. Besides, the membrane contains a variety of ion channels essential for osmotic balance regulation and for signaling by exchange of ions [16,17,18,20]. Some of these channels are linked to deformation by mechanical sensation [21,22]. It is known that the density of human RBCs increases with their age [23,24] during the average lifetime of 115 to 120 days [25]. Therefore, in principle, centrifugation of RBCs in a density gradient allows for sorting by age. A rapid method for the separation of RBCs in age-dependent fractions was described in 1980 [26]. This led to a variety of studies on the relation of morphological and biochemical parameters to cell age. For instance, the deformability of RBCs declines during the aging process [27]. These investigations on relating cell age to other cellular properties are important for RBCs in particular. Reticulocytes enter the circulation in an enucleated state and thus are void of a protein transcription and translation machinery. This means RBCs experience aging without any renewal processes, which is a unique behavior [28].

Lutz et al. found the RBC density to be a reliable indicator for cell age [29]. Unfortunately, the biochemical markers for RBC age populations are very sparse. They include remnants of RNA or, to some extent, mitochondria in reticulocytes and the transferrin receptor (CD71) for the youngest population of the reticulocytes. Furthermore, reticulocytes can be sorted, for example, by fluorescence associated cell sorting (FACS) based on the above-mentioned markers. Then, the RBC can be stained with in vivo compatible markers, such as PKH dyes. After transfusion of the stained RBCs, it is possible to get age-defined RBCs [30,31].

When leaving the single cell level and considering cell populations, the membrane protein 4.1 provides a reliable measure for cell age. While the total amount stays constant during the lifetime of an RBC, the ratio between its forms 4.1a and 4.1b increases during RBC aging [32]. This is the result of a time-dependent, non-enzymatic deamidation of an asparaginyl residue in protein 4.1b [33]. Therefore, the protein 4.1a/4.1b ratio can be regarded as a molecular clock [34]. Measurements of the protein 4.1a/4.1b ratio can be used to determine the average age of an RBC population independent of other physiochemical parameters such as cell density [35].

The distribution of RBCs after centrifugation in a self-forming Percoll gradient is surprisingly not homogeneous but characterized by a heterogeneous structure of discrete bands [26,29,36,37,38]. Lutz et al. observed a redistribution of cells extracted from the gradient. Therefore they concluded that a uniform density of cells from a particular fraction in the gradient is not guaranteed. Additionally, they suspected contamination of dense cells in light fractions that reflected in the protein 4.1a to 4.1b measurements and considered aggregation as a possible reason [29].

Recently, a quantification of the band patterns by image processing was investigated for sickle cell anemia using graph convolutional networks [39], and the applicability in diagnostics was discussed. Indeed, band patterns in Percoll gradients have the potential to serve as diagnostic or even prognostic markers [37,39], admitting that a full causal understanding is not always given and the application of artificial intelligence is an extremely useful tool for implementation [40]. Still, the formation of the discrete bands and their patterns is a phenomenon that could so far not be explained [41] and is investigated in this paper. 

## 2. Materials and Methods

### 2.1. Blood Collection, RBC Preparation, and Solutions

Blood was collected from healthy donors into EDTA or heparin tubes by venipuncture, washed, and resuspended. It was previously shown that the anticoagulant had no significant effect on the band structure formation in Percoll gradients [38]. For microscopic aggregation measurements, blood was collected by finger prick. The blood collection was performed following the declaration of Helsinki and was approved by the ethics committee of ‘Ärztekammer des Saarlandes’, permit number 51/18. For centrifugation, either whole or washed blood was used. Washing of full blood samples was carried out at 1700 to 2000×g for 5 to 7 min in 1.5 mL tubes prior to Percoll gradient centrifugation, or at 1300×g for 4 min in 1.5 mL Eppendorf tubes prior to microscopy. In order to remove residual Percoll particles, we washed samples that were extracted from gradients at 4000×g for 7 min. For washing and resuspension, Phosphate Buffered Saline (PBS) diluted from a 10× concentrated stock solution or from an undiluted commercial stock (Gibco 100010-031, Thermo Fisher Scientific, Waltham, MA, USA), and alternatively *Chur*-solution, containing (in mM): 140 NaCl, 4 KCl, 0.75 MgSO_4_, 10 glucose, 0.015 ZnCl_2_, 0.2 glycine, 0.2 glutamate, 0.1 arginine, 0.6 glutamine, 0.2 alanine and 20 HEPES imidazole, pH 7.4, as previously described [36], were used. Hypotonic buffers were prepared by dilution of isotonic solutions. All solutions were prepared with MilliQ water. Osmolality was checked using freezing point osmometers (Osmometer Automatic, Hermann Roebling Messtechnik, Berlin, Germany). After washing, RBCs were fixed by resuspending them at 5% hematocrit (Ht) in a 1% glutaraldehyde solution [42]. During pipetting, the flow velocity was kept low to avoid shear stress and the sample was incubated at room temperature (RT, between 21 °C and 25 °C) for 20 min on a tube roller.

### 2.2. Percoll Density Preparation, Centrifugation, and Sub-Band Extraction

**Figure 1:**
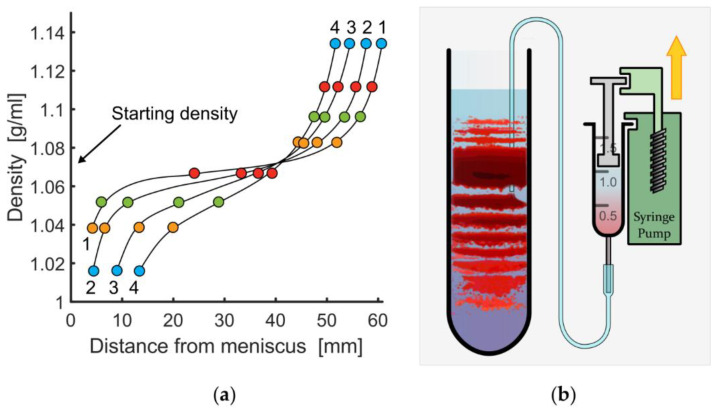
(**a**) Density gradient measurements of Percoll medium by *GE Healthcare* using colored beads in an angle head rotor at 20,000× *g* and varying centrifugation duration, (1) 15 min, (2) 30 min, (3) 60 min, (4) 90 min, reproduced from [5]; (**b**) scheme of the cell-extraction process of from a single band using micro medical tubing and a syringe pump.

Commercial medium, Percoll or Percoll Plus (17-0891-01 or 17-5445-01, GE Healthcare, Chalfont St Giles, Buckinghamshire, UK), was diluted to obtain a density matching the average cell density at a given hydration state. Percoll Plus did not reveal any differences in band formation over Percoll. Density distributions for selected centrifugation times are presented in Figure 1a. The average cell density was obtained from a centrifugation series in isotonic Percoll media of different densities; the diluent was a concentrate of PBS or *Chur*-solution; the concentration was adjusted to obtain the desired tonicity and medium density. The pH was adjusted to 7.4 using HCl and NaOH. Centrifugation was carried out in a *Hermle Z36 HK* equipped with rotor *221.22* (Hermle Labortechnik GmbH, Wehingen, Germany), a *Sorvall Lynx 4000* with rotor *A22-24 × 16* or *Sorvall RC 5B Plus* with rotor *SS-34* (Thermo Fisher Scientific, Waltham, MA, USA), or an *Optima XPN-80* with rotor *SW 32 Ti* (Beckman Coulter, Brea, CA, USA), at 25 °C or 34 °C and 20,000×g for 20 or 30 min. For band extraction from inside the distribution, a syringe pump, 40 μL/min, with microfluidic tubing, 0.9 mm inner diameter and 1.3 mm outer diameter, was used as outlined in Figure 1b. Sub-band extraction was carried out from the top using a syringe with a hypodermic needle, 0.8 mm×120 mm. Extracted cells were washed and resuspended to remove Percoll residue.

### 2.3. Microscopy and Image Analysis

Aggregation measurements were based on microscopic bright field images from an *Eclipse TE2000* (Nikon, Tokyo, Japan) equipped with a *DMK 33UP5000* (The Imaging Source Europe GmbH, Bremen, Germany) and a *CFI Plan Fluor DL 10*× *NA 0.3* (Nikon, Tokyo, Japan). Homogeneous RBC suspensions in isotonic Percoll media of different concentrations were placed in a μ*-slide* with 18 wells (ibidi GmbH, Munich, Germany), 5 mm in diameter and a volume of 30 μL each; sealed after complete filling. Images were taken after sedimentation for 24 h at RT. Cell counts were obtained using a cell counting chamber (*Malassez* 0.2 mm, Paul Marienfeld GmbH, Lauda-Königshofen, Germany or *Bright Line Counting Chamber* 0.1 mm, Horsham, PA, USA) and bright field microscopy.

### 2.4. Band 4.1 Protein Deamidation Detection by Western Blot

Sample preparation: One volume of an RBC suspension at 2% or 20% Ht was mixed with 9 volumes of “sample buffer” (18.2 mM Tris-HCl, pH 6.8, 5% SDS (*w*/*v*), 1.9 mM EDTA, 13% (*w*/*v*) sucrose, 40 mg/L bromophenol blue as a tracking dye, 70 mM dithiothreitol added fresh before use). The sample was incubated at 60 °C for 15 min then stored frozen in small aliquots (approximately 100 μL each) so that only the amount required for loading the gel was thawed before use.

A *Mini-Protean 3* system (Bio-Rad Laboratories Inc., Hercules, CA, USA) was used to cast the gels and perform gel electrophoresis. A stacking gel was layered on top of a separating gel, also called running gel. Constituents for the running gel were, in (*v*/*v*), 23.3% of a 30% (*w*/*v*) acrylamide solution in water, 25% of 1.5 M Tris/HCl pH 6.8, 1% of 10% (*w*/*v*) SDS, 1% of 10% (*w*/*v*) ammonium persulfate (APS), 2.5% of 1% (*w*/*v*) Temed, 47.2% H_2_O. The stacking gel was less dense, and contained, in (*v*/*v*), 10% of a 30% (*w*/*v*) acrylamide aqueous solution, 10% of 1.25 M Tris/HCl pH 6.8, 1% of 10% (*w*/*v*) SDS, 1% of 10% (*w*/*v*) APS, 10% of 1% (*w*/*v*) Temed, 68% H_2_O. Polymerization started after the addition of Temed and APS. During polymerization, a comb was inserted into the stacking gel to create wells (10 or 15) for loading the samples, usually 10 μL of cell lysate per well. In one well, a sample of pre-stained molecular weight standard proteins was loaded (Precision Plus Protein™ All Blue, code 1610373, Bio-Rad Laboratories Inc., Hercules, CA, USA). After loading the samples, the electrophoretic run was conducted at constant voltage (100 V) until proteins reached the running gel and at 150 V constant during the separation in the separating gel. In order to further separate the 4.1a and 4.1b bands, we overrun the gels by 30 min after the tracking dye reached the bottom of the gels. The stacking gel was removed. Protein transfer made use of semidry electroblotting (*Trans-Blot*, Bio-Rad Laboratories Inc., Hercules, CA, USA) for transferring the proteins to a PVDF membrane (0.2 μm pores). After the transfer, the membrane was incubated with a “blocking” solution made of 5% skimmed milk, 20 mM Tris, pH 7.4, 150 mM NaCl and 0.05% Tween-20 (*v*/*v*). After the “blocking”, the membrane was washed two times with a “washing buffer” [50 mM Tris/HCl, pH 7.5, 200 mM NaCl, 1 g/L polyethylene glycole (PEG)-20000, 0.5 mL/L Tween-20, 1 g/L bovine serum albumin (BSA)]. The membrane was then incubated overnight at 4 °C under gentle rocking with a 1:1000 dilution in washing buffer of a mouse monoclonal antibody against protein 4.1R (primary antibody: clone B-11, code sc-166759 Santa Cruz Biotechnology, Dallas, TX, USA). The primary antibody solution was removed and the membrane subjected to 8 washes, 8 min each, with washing buffer at RT, and then incubated for 1 h with a secondary antibody (HPR-conjugated, goat anti-mouse IgG, code 170-6516, Bio-Rad Laboratories Inc., Hercules, CA, USA). After removing the secondary antibody, the membrane was washed 6 times for 6 min each and it was then treated with the chemiluminescence reagent: equal parts of peroxide and luminol (*Amersham ECL*, Cytiva, Marlborough, MA, USA) for 3 min. A *ChemiDoc XRS+* (Bio-Rad Laboratories Inc., Hercules, CA, USA) was used for acquiring the chemiluminescence signal. Data was analyzed by our own algorithms using the *Bio-Formats* library [43] for *Matlab*^®^.

### 2.5. Statistical Analysis

In order to assess significance in the difference of two data sets, we employed the two-sample t-test for equal means. A p-value below 0.05 was considered significant and marked with a star, values smaller 0.01 with two stars. In bar charts, the standard error of mean (SEM) was computed by dividing the standard deviation (SD) by the square root of the number of data points. SDs were computed from the unbiased sample variance. In box plots, quantiles are computed using sorting algorithms and linear interpolation; notches give an estimate for the 95% confidence interval (CI) from the interquartile range (IQR) by [44]:

(1) $$95\%{\mathrm{CI}}_{\mathrm{mean}}=\mathrm{median}\pm \frac{1.57\times \mathrm{IQR}}{\sqrt{N}}$$

for a sample size of N. No overlap of notches indicates a *p*-value < 0.05. If not stated otherwise, whiskers indicate 1.5 times the interquartile range. Kernel density estimation (kde) was utilized to compute probability density functions (pdf). The optimal bandwidth was estimated by a rule of thumb, $h_{\mathrm{opt}}=1.06\;\mathrm{SD}\;N^{-1/5}$. In aggregation measurements the accessible parameter was the area of connected absorption regions in brightfield images. It was assumed, that those regions primarily originate from projections of cell aggregates. Sources of distortion are the orientation of aggregates and stacking of disconnected cells or cell aggregates. Statistics was carried out on the area of connected projection regions.

## 3. Results

It was absolutely unclear why Percoll centrifugation of RBCs does not provide a continuous profile but rather distinct bands [41] albeit the self-forming Percoll gradient is continuous (Figure 1a, [5]). From all we know about RBC aging [28] a discrete aging process is unlikely to be the cause of the discrete RBC bands.

### 3.1. Variation of Basic Experimental Parameters

**Figure 2:**
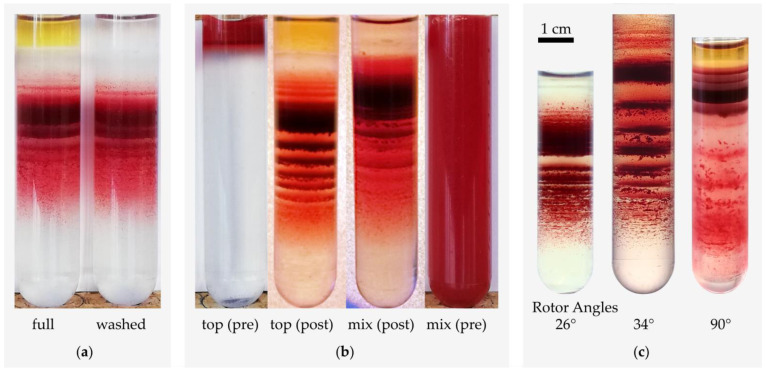
RBCs in self-forming continuous Percoll gradients under various experimental conditions. The scale bar is valid for all panels. (**a**) Distributions of a whole blood sample (**left**) and a washed RBC suspension after simultaneous centrifugation; the samples were loaded on top of the respective Percoll suspension before centrifugation. (**b**) Appearance, before and after centrifugation, of a sample of whole blood that was layered (**left**) on top of the Percoll medium, or homogeneously mixed with it, prior to centrifugation (**right**). In (**a**,**b**) heparin was used as the anticoagulant at blood withdrawal, and the centrifugation conditions were 20,000× *g* at 34 °C for 30 min. (**c**) Rotor heads of different angles were used; 90° belongs to the swing-out rotor. Blood samples in (**c**) were different, but all from healthy donors; 34° with heparin, others with EDTA; centrifugation conditions were 20,000× *g* for 20 min at 25 °C. The different centrifuge models hold tubes different in size and material. This could influence the gradient shape and band formation.

In a first approach, we aimed to test the influence of very basic experimental parameters on the band formation *per se* or the band pattern in order to get an initial glimpse of the nature of the band formation. Considering that RBCs form a percolating gel in blood plasma [45,46,47,48], we wondered if there is a difference in whether whole blood or washed RBCs are added. Figure 2a shows that there are no major differences between the band pattern of the whole blood sample and that of the washed RBCs sample. Next, we wondered if the starting conditions make a difference, whether the blood is layered on top of the Percoll or if RBCs and Percoll are mixed thoroughly. Figure 2b presents the tubes before and after centrifugation and indicates differences in the band patterns, but still shows discrete band formation with the main population at a similar height. It indicates that the initial position of the RBCs influences the band pattern but in principle does not prevent or favor its formation.

Furthermore, we considered the influence of the direction of the centrifugation force (different from the angle of the tube in the rotor) and its superposition with the gravitational force as possible determinants of the discrete bands. Such forces could induce, for example, convection flow [49] and thus influence pattern formation. To this end, we performed Percoll centrifugations with different rotors as outlined in Figure 2c. Similar as to Figure 2b, we detected slight differences in the band patterns but no prevention of the formation effect.

### 3.2. Recentrifugation of Particular Bands

**Figure 3:**
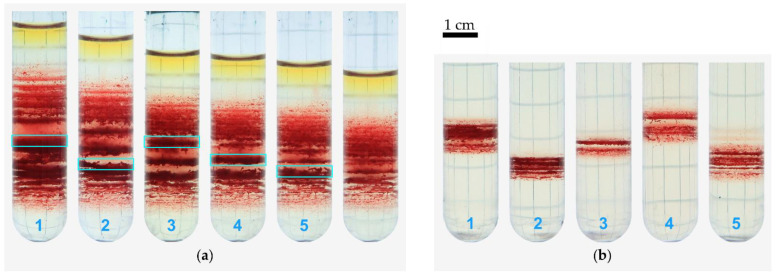
Bands were subsequently extracted from the distribution of a full blood sample (**a**), using a syringe pump, compare to Figure 1b. Each succeeding image in (**a**) shows the tube after extraction of the labeled layer. In a second step, the collected cells were layered on top of another Percoll suspension of identical composition as in the first centrifugation and centrifuged under equivalent conditions (**b**). Band 3 appears lower due to a thinning caused by a larger buffer volume. The scale bar is valid for both panels.

**Figure 4:**
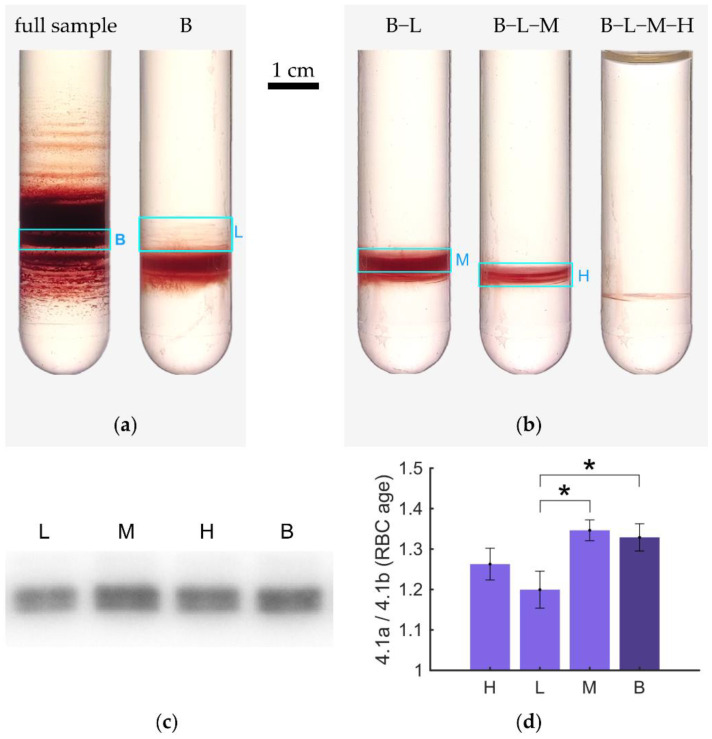
Western blotting after redistribution of an extracted band. Panel (**a**) shows the distribution of a full blood sample from heparin tubes after centrifugation in an isotonic medium. A band (B) was extracted and redistributed in an equivalent gradient. (**b**) From the recentrifuged tube, a low (L), a medium (M), and a high (H) density layer were extracted successively. Panel (**c**) shows an excerpt of a western blot. RBCs were treated with the sample buffer as cell suspensions at 20% Ht, and 10 μL of each sample were loaded in the gels. In (**d**), bar graphs of the mean 4.1a to 4.1b ratio derived from six samples for each band, randomly distributed over four different gels, error bars show the SEM; a single star indicates p<0.05.

To get a better insight into the bands, we extracted some as outlined in Figure 3a, resuspended, and centrifuged them under equal experimental conditions (same Percoll medium, same centrifugation force, same duration). The results are shown in Figure 3b. All distributions show the appearance of new bands. Please note that this is not just a higher magnification but the appearance of new bands, which again leads to a discrete distribution. In another experiment, we aimed to investigate the age structure of the bands and sub-bands. To this end, we extracted one band from a Percoll gradient and resuspended this sample in the same Percoll medium, and centrifuged it a second time as indicated in Figure 4a. From this re-centrifugation, we extracted three bands of low (L), medium (M), and high (H) density, as indicated in Figure 4b for further analysis. Based on these extractions we performed western blots (Figure 4c) to determine the protein band 4.1b to 4.1a ratio, which can be regarded as a molecular clock [35].

The statistical analysis (performed as outlined in Appendix A) is shown in Figure 4d and indicates significant differences between both the original band B (average) and sub-band L, and between the sub-bands M and L. Non-significant differences were detected between sub-bands (H vs. L or H vs. M) and between the original band B and sub-band M (obvious) but also between the original band B and sub-band H.

### 3.3. Percoll-Induced Cell Aggregation

**Figure 5:**
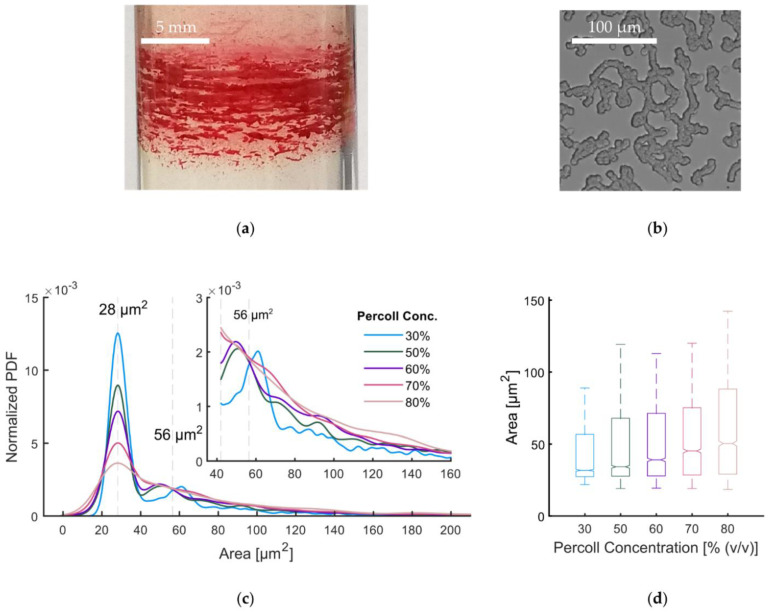
Aggregation of RBCs in a Percoll medium: (**a**) macroscopic aggregates in the band pattern after centrifugation; (**b**) *Rouleaux* formation of RBCs extracted from a gradient under the microscope; (**c**) distributions of the projection area as measured from microscopic images for different Percoll concentrations; (**d**) boxplots of the distributions in (**c**), edges show quartiles, whiskers the 2.5th and 90th percentiles, notches the estimated 95% confidence interval.

A possible explanation for a compromised density separation is that not individual RBCs are density resolved, but RBC aggregates instead. Already by eye, RBC aggregates are visible at the edges of the bands, as it is exemplified in the enlargement in Figure 5a.

We used bright field microscopy to check if RBCs in Percoll form similar aggregates (stack of coins or *rouleaux*) as is known from blood plasma and dextran solutions [50]. Figure 5b shows that this is indeed the case. A more detailed analysis of the projection area of aggregates in dependence on the Percoll concentration was derived from images as depicted in Figure 5b. The probability density function is plotted in Figure 5c and box plots of the aggregate projection area for various Percoll concentrations are given in Figure 5d.

All distributions show a maximum at $28.2{\;\mu m}^{2}$ ± $5.4{\;\mu m}^{2}$. This maximum can be assigned to non-aggregated single cells. A second peak is present around $56\;{\mu m}^{2}$ originating in aggregates with two cells, respectively. The second peak shifts towards smaller values with increasing Percoll concentration. This indicates a more compact binding. Peaks of larger aggregates are expected to be less prominent due to the variety of three dimensional configurations leading to different projection areas. The single cell peak decreases in height and area with increasing Percoll concentration. This means, aggregates increase in size and cell number. Although this RBC aggregation provides an explanation of compromised density separation, the formation of individual bands is still elusive.

### 3.4. Mathematical Modelling of the Sedimentation Process

**Figure 6:**
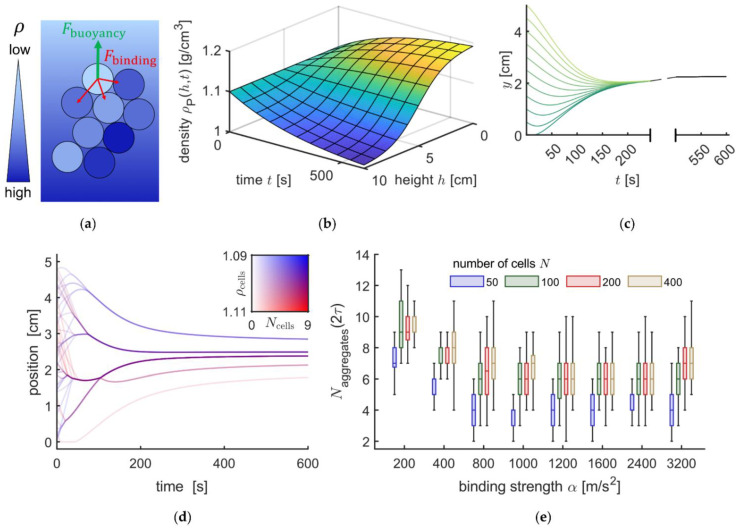
Mathematical description of aggregation during sedimentation in a density gradient. (**a**) Schematic illustration of an aggregate in a density gradient, the opacity of blue indicates the density; (**b**) graph of the time and space dependent density function; (**c**) simulation results for a single cell with density 1.102 g/mL and different initial positions; (**d**) sedimentation curves for 50 cells interacting via a contact potential, the amount of red corresponds to the density, the opacity to the number of cells in an agglomerate; (**e**) distributions of the number of aggregates in the final configuration, i.e., at twice the gradient time constant τ, for different cell numbers and binding strengths.

We developed a theoretical model to simulate the forces acting on RBCs during centrifugation. The forces acting on an RBC in an aggregate are schematically illustrated in Figure 6a and the spatiotemporal formation of the Percoll gradient as used in the model is plotted in Figure 6b. The full model is described in Appendix B.

Figure 6c shows an initial validation of the model. Independent of the starting point an individual RBC (density of 1.102 g/mL) would always end up in the same position at the end of the centrifugation process. However, if the model computes the motion of 50 RBCs of various densities, we end up with five discrete bands as outlined in Figure 6d. We performed a variety of simulations with different binding strengths (from $\alpha =200\;m/s^{2}$ to $3.2\;\mathrm{km}/s^{2}$), and RBC numbers up to 400 as summarized in Figure 6e. We deduced conditions that lead to the formation of aggregates and their stability in the equilibrium distribution after centrifugation. We observed that the RBC distribution is characterized by bands consisting of RBC aggregates.

### 3.5. Experimental Conditions Suppressing RBC Aggregate Formation

**Figure 7:**
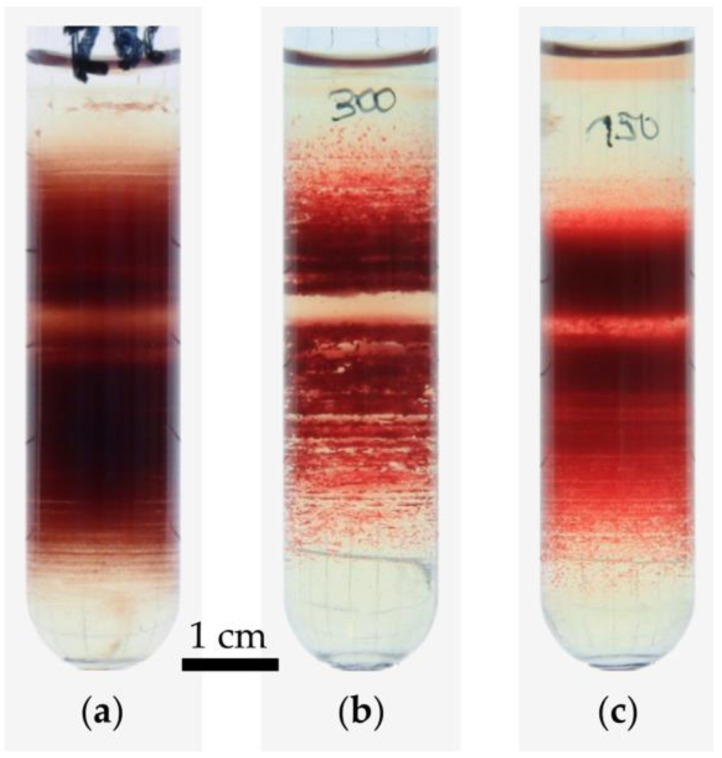
Suppressing aggregation. (**a**) Isotonic RBCs fixed with GA; (**b**) an isotonic control sample; (**c**) a hypotonic sample of 150 mosmol/kg H_2_O; the density medium was adjusted to account for hydration-related changes in cell density, centrifugation conditions were 25 °C, 20,000× *g*, 20 min in a *Hermle Z36 HK*. EDTA was used as the anticoagulant at blood withdrawal.

If RBC aggregation is the cause of both, the band formation and the impaired age separation, suppression of the aggregation should reveal support of the model described above and refinement of the age-separation method, respectively. One method was the fixation of RBCs using glutaraldehyde (GA). Since GA fixation is associated with cell shrinkage [42], centrifugation of fixed RBCs required an adaptation of the Percoll gradient. The result is shown in Figure 7a in comparison to fresh RBC centrifugation (Figure 7b). It clearly shows that GA fixed RBC are distributed much more homogeneously, while a high number of thin bands is formed. Another option to prevent aggregation would be to swell RBCs in order to reduce the RBC interaction surface compared to discocytes. The easiest way to transform normocytes into spherocytes (or stomatocytes of type III) [7] is to place them in a hypotonic solution. In addition, this approach required an adaptation of the Percoll gradient. The result is presented in Figure 7c and mirrors the results of the GA-fixed RBCs.

### 3.6. RBC Age Determination in Hypotonic Solutions

**Figure 8:**
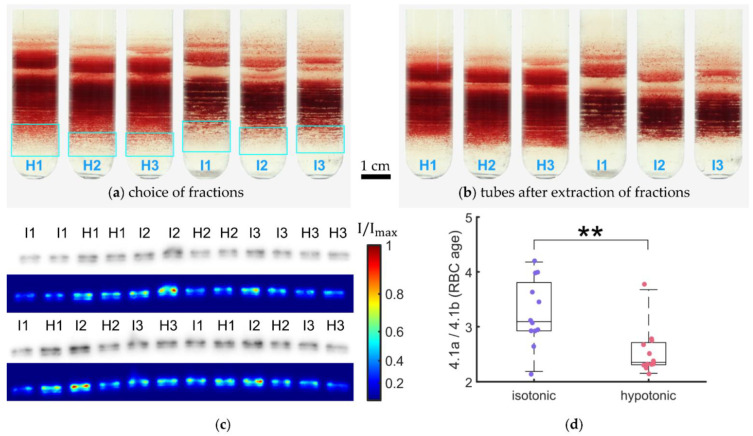
Age distribution of the densest cells population. (**a**) Hypotonic (H) and isotonic (I) samples (blood withdrawn in EDTA) were centrifuged in density matched Percoll media in a *Hermle Z36 HK* at 25 °C. From each of the distributions, 1 mL of suspension was extracted from the regions marked with a blue rectangle. Panel (**b**) shows the same tubes after extraction; (**c**) western blots of the RBC samples (a 2% Ht RBC suspension was treated with the sample buffer for gel electrophoresis, as described in Materials and Methods), raw images, and logarithmic false-color representations. The logarithmic false-color version emphasizes intensity differences between the pair of bands. (**d**) The protein 4.1a to 4.1b ratio was computed and the statistical results are plotted. Whiskers show the 5th and 95th percentiles. Average protein ratios differ significantly: p=0.0037 (**).

The related question is if the more homogeneous RBC distribution also leads to a better/sharper age separation. Since age separation is performed for functional RBC tests, the separation of fixed RBCs is of lower interest and we followed a systematic investigation of hypotonic swollen RBCs. To avoid any difficulties in band recognition between isotonic and hypotonic Percoll solutions it would be advantageous to choose either the uppermost or the lowest fraction. Due to the terminal density reversal phenomenon [51], we aimed for the analysis of the lowest/densest cells. Figure 8a,b show the Percoll centrifuged samples before and after extraction of the densest layer, respectively. Four samples of each layer were run on two different gels and western blots for Protein 4.1 was performed as depicted in Figure 8c. Densiometric analysis of the western blots as described in Appendix A revealed a significant difference between in Protein 4.1a to 4.1b ratio for the densest cells in isotonic and hypotonic Percoll solutions (Figure 8d).

## 4. Discussion

We start the discussion with the discrete band formation of RBCs in a continuous (self-forming) Percoll gradient. This band-formation should not be mistaken for the (also often employed) Percoll layer preparation, e.g., in [52], where pre-prepared Percoll media of decreasing density are layered carefully, RBCs are placed on top and centrifuged for a shorter time at lower centrifugal forces compared to the self-forming gradient centrifugation. While the pattern of these layers seems to be characteristic for certain pathologies [39] and may reflect severity of the hereditary diseases such as spherocytosis [37], its origin was a mystery so far [41]. We confirmed that the band pattern is reproducible for particular blood samples and does not depend on whether RBCs are added in autologous plasma or washed in phosphate or *Chur* buffer (Figure 2a). The reason is most probably that already at the very beginning of the centrifugation process, RBCs leave the plasma/PBS phase into the Percoll solution and from that point onwards there is no difference between previously isolated and washed RBCs.

However, results from different initial cell distributions indicate that the dynamics during the sedimentation influence the band formation (Figure 2b). This is evidence for the contribution of other parameters than the intrinsic single cell properties. The reproducibility of band structures emerging from the same initial distribution suggests that the resulting distribution is deterministic. That means it depends on the initial conditions and the centrifugation parameters only. In other words, it is not a random result. Different initial conditions can lead to different but deterministic and reproducible results. In our microscopic model, the prerequisite for aggregation of two cells is a crossing or tangency of their trajectories leading to proximity and the onset of short range interactions. The aggregation process depends on the number of those approaches in the sample and the (local) sample density. Therefore it is influenced strongly by the initial distribution.

We were suspicious about ‘non-ideal’ forces in the centrifugation process, such as differences in centrifugation forces between the opposite sides of the centrifugation tubes in fixed angle rotors or vector differences between gravitation force and centrifugation force as putative causes for the band formation. However, comparing the results of different rotor geometries (Figure 2c) just reveals that the distribution width of the bands increases with increasing rotor angle due to the less pronounced sigmoidal density distribution of the Percoll [5]. The band formation *per se* was conserved in all rotor geometries.

To further investigate the nature of the bands and their formation, we extracted bands and recentrifuged them a second time under otherwise identical conditions. This resulting in a broadening of the spatial cell distribution and the formation of new sub-bands reflecting a broader distribution of both, RBC density (Figure 3) and RBC age (Figure 4). These properties are in agreement with the effect of RBC aggregation as proposed by Lutz et al. [29] and as confirmed by our microscopic investigation (Figure 5).

While the *rouleaux* formation can explain the broadening of the RBC distribution when recentrifuged as well as impaired age-density correlation, it still does not explain the band formation. To this end the mathematical model of the sedimentation process as outlined in Figure 6 and Appendix B provides a possible explanation. The numerical investigations of the time-dependent sedimentation and aggregation process with randomized initial conditions in a simplistic model supported the established equilibrium conditions. Moreover, a statistical insight in the numerical solutions showed that the final distributions are characterized by a low number of cell aggregates compared to the number of cells. Furthermore, the average number of aggregates became independent of the interaction force above $80\times 10^{3}\;\mathrm{cm}/s^{2}$ and cell number for N>50. The critical force of 80 pN, assuming a cell mass of $10^{-13}\;\mathrm{kg},$ is comparable to previous measurements of depletion forces [53]. Experimental observations of the band structure of RBC sedimentation distributions in Percoll suggest an independence on statistical processes. The resulting distribution is reproducible to the eye given the same initial conditions and centrifugation parameters. Hence, the band structure can be described as deterministic with respect to those influences. Suppression of RBC aggregation would hence decrease band formation which could indeed be shown for two independent approaches, osmotic swelling of RBCs (Figure 7c) and RBC fixation by GA (Figure 7a).

We aimed for an initial transfer of the gained knowledge towards a more precise RBC age separation by preventing RBC aggregation and hence band formation. The idea was to centrifuge the cells in a hypoosmotic Percoll solution and proof the improved age separation by a protein band 4.1a to 4.1b analysis vs. isotonic preparations. We indeed achieved a significant difference for the densest RBC population (Figure 8d), but the result was opposite to our expectations. As we have less aggregation in hypotonic solutions, we expected less ‘contamination’ by younger cells and therefore an older population with a higher band 4.1a/4.1b ratio. This illustrates nicely that age-dependent effects induced by hypoosmotic ‘stimulation’ of the RBCs overwrites the effect of the aggregate prevention. These hypoosmotically induced effects could be the activation of the K^+^/Cl^−^-cotransport in reticulocytes [54,55], which is likely to shrink them, or the unequal activation of mechanosensitive channels such as Piezo1 [56,57] in RBCs of different age, resulting in a heterogeneous Ca^2+^ uptake, which, in turn, will again lead to cell dehydration and shrinkage through activation of the Ca^2+^-sensitive-K^+^ channel (Gárdos channel) and loss of KCl and water [58]. It demonstrates clearly that maneuvers that stimulate (directly or indirectly) changes in ion homeostasis and hence on the hydration state (density) of the RBCs disrupt the direct correlation between buoyant density and age and compromise the density-based age separation of RBCs [30,59]. Also, the hypoosmotic stress could have caused a slight increase in hemolysis among the oldest cells altering their abundance (less than 1%). A sign of a partial hemolysis is a reddish supernatant (see, e.g., Figure 7c).

## 5. Conclusions

We could provide a physical explanation for the RBC band formation in continuous Percoll density gradients based on RBC aggregate formation. This aggregate formation compromises the density based age separation. Suppressing aggregation by osmotic swelling has a more severe effect on RBC density, compromising age separation to a higher degree.

Further research is required to modify or improve the density centrifugation process to better represent the RBC age distributions. In principle a decrease of aggregability should lead to a more homogeneous distribution.

## Figures and Tables

**Figure A1:**
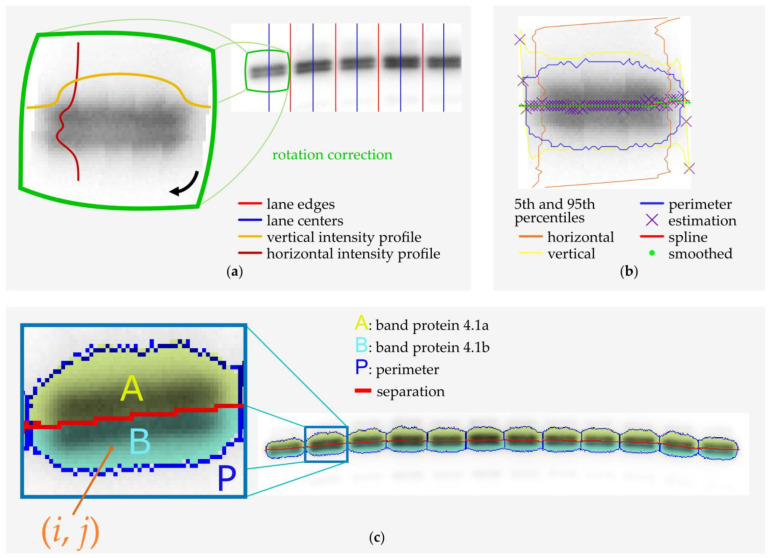
Scheme of the automated image analysis algorithm. (**a**) Intermediate edges and lane positions are detected, each pair of bands is isolated and rotated for alignment; horizontal and vertical intensity profiles are extracted; a double-gaussian is fitted to the latter. (**b**) The 5th and 95th percentiles of the profiles along each axis are determined and combined to compute a perimeter; separation points are estimated from the double-gaussian. A spline regression is carried out to discard outliers; a smoothed discrete edge is deduced. From (**b**,**c**) an inverse coordinate transformation is applied to fit the perimeter and separation edge on the raw data.

## Data Availability

The data presented in this study are available on request from the corresponding author.

## Appendix A

A series of image with increasing exposure time are acquired in the *ChemiDoc XRS+.* Of each series the image is chosen automatically which maximizes contrast without showing saturation. The raw camera image provides a resolution of $520\times 696\;{\mathrm{px}}^{2}$, 350 ppi respectively, and 16 bit intensity depth. An algorithm was designed to perform an automated measurement of the protein 4.1b to 4.1a ratio which is given by the integrated photoluminescence intensity as summarized in Figure A1. In order to work with the raw data we used the Bio-Formats library [43]. Sample lanes are separated by slicing the image vertically at intermediate positions. Lane centers are determined. All those positions are measured from intensity profiles utilizing peak detection algorithms. A coordinate transformation makes the analysis independent of lane orientation. The 5th and 95th percentiles in the horizontal and vertical intensity distributions are determined for each image row and column, respectively. By means of a gray value matching, a perimeter (P) around both protein bands (A and B) is derived. The intensity on the perimeter gives an approximation for the background signal. Protein 4.1 bands are separated. In order to do so, we perform a column-wise regression of a double-gaussian. The cross-over position $s_{j}$ in the in the *j*th column is determined using two different methods. Method A involves numerically finding the minimizer of the condition for a local minimum.

(A1) $$s_{j}=\underset{x}{\min}\left(\left|\frac{a_{1}\left(x-b_{1}\right)}{c_{1}^{2}}\exp\left(-\frac{{\left(x-b_{1}\right)}^{2}}{c_{1}^{2}}\right)+\frac{a_{2}\left(x-b_{2}\right)}{c_{2}^{2}}\exp\left(-\frac{{\left(x-b_{2}\right)}^{2}}{c_{2}^{2}}\right)\right|\right)$$

The turning point position is estimated in method B by minimizing the second derivative.

(A2) $$s_{j}=\underset{x}{\min}\left(\left|\frac{a_{1}}{c_{1}^{2}}\left(1-\frac{2{\left(x-b_{1}\right)}^{2}}{c_{1}^{2}}\right)\exp\left(-\frac{{\left(x-b_{1}\right)}^{2}}{c_{1}^{2}}\right)+\frac{a_{2}}{c_{2}^{2}}\left(1-\frac{2{\left(x-b_{2}\right)}^{2}}{c_{2}^{2}}\right)\exp\left(-\frac{{\left(x-b_{2}\right)}^{2}}{c_{2}^{2}}\right)\right|\right)$$

A smoothing spline is fitted to the separation points $\left(j,s_{j}\right)$. The resulting separation line encloses each band of the 4.1 pair together with the perimeter. An intensity summation over each band (A and B) is then carried out. The resulting integral intensities are corrected for background signal and divided to compute the protein ratio.

(A3) $$I_{0}=\frac{\sum_{\left(i,j\right)\in P}I\left(i,j\right)}{\sum_{\left(i,j\right)\in P}1}\;,$$

(A4) $$\frac{c\left(4.1a\right)}{c\left(4.1b\right)}=\frac{\sum_{\left(i,j\right)\in A}I\left(i,j\right)-\sum_{\left(i,j\right)\in A}I_{0}}{\sum_{\left(i,j\right)\in B}I\left(i,j\right)-\sum_{\left(i,j\right)\in B}I_{0}}\;.$$

Our algorithm is robust and provides a good separation also in case of distorted bands.

## Appendix B

A theoretical model was established to describe the sedimentation process of RBCs during centrifugation in a self-forming gradient. The gradient formation was assumed to be an external input function independent of the presence of cells. The density of the suspension medium was assumed to be merely a function of height in the tube *y* and time *t*, to be constant initially, and to transition into a sigmoidal equilibrium shape according to measurements, see Figure 1a. This transition was assumed to be an exponential time relaxation process. A function meeting those requirements is given by

(A5) $$\rho_{P}\left(y,t\right)=\left\{\rho_{P,0}+\epsilon \;\log\left(\frac{\lambda }{y-h_{0}}-1\right)\right\}\times \left\{1-\exp\left(-\frac{t}{\tau }\right)\right\}+\rho_{P,0}\;\exp\left(-\frac{t}{\tau }\right)\;,$$

where $\rho_{P,0}=1.1\;g/\mathrm{mL}$ is the initial homogenous density, τ=300 s the time constant of the gradient formation, λ=52 mm the characteristic length, ϵ=0.0122 g/mL the density spread, and $h_{0}=-1\;\mathrm{mm}$ a height shift to center the gradient at $\lambda /2+h_{\;0}=25\;\mathrm{mm}$. This translates into a tube length of 50 mm with a central density saddle. The parameter values were obtained from a non-linear regression to experimental data. The function fulfills mass conservation at any time.

We set up the equation of motion for the vertical position of the *i*th cell assuming azimuthal symmetry and including the centrifugal force, a buoyancy term, Stokes’ friction, and a short range contact interaction.

(A6) $${\ddot{y}}_{i}=\frac{\rho_{P}\left(y_{i},t\right)-\rho_{\mathrm{cell}}^{i}}{\rho_{\mathrm{cell}}^{i}}\;\mathrm{rcf}-\frac{9\eta }{2\rho_{\mathrm{cell}}^{i}{\bar{R}}^{2}}\dot{y_{i}}+\overset{N}{\underset{j=1}{\sum }}\alpha \mathrm{sign}\left(y_{j}-y_{i}\right)\left[\Theta \left(R_{\max}-\left|y_{i}-y_{j}\right|\right)-\Theta \left(R_{\min}-\left|y_{i}-y_{j}\right|\right)\right]\;.$$

The viscosity of the suspension medium was η=15 mPa s, $\bar{R}=5\;\mu m$ the mean cell radius, $R_{\min}=4\;\mu m$ the minimal interaction range, and $R_{\max}=20\;\mu m$ the maximal, respectively. The binding strength α is a mass specific force (i.e., an acceleration). The total number of cells N and the binding strength α were varied, the resulting distributions at t=2τ studied. The relative centrifugal force (rcf) was set to 20,000×g.

The equations of motion were solved using an implementation of the four-order Runge-Kutta method (RK4) in C++ with a step size of dt=1 μs. The initial condition was a randomized homogenous distribution in height and gaussian distribution in density with central density of 1.1 g/mL and standard deviation of 4 mg/mL, as found in experiments for physiological conditions [60,61]. We derived equilibrium conditions from the equations of motion.

- The displacement force (buoyancy) that includes acceleration of cell volume and liquid volume must be equal to the sum of binding forces for each cell.
- The average of all cell densities in an aggregate equals the suspension density at the average position of those cells.

Our numerical results supported those conditions down to numerical errors.

## References

[1] Fahraeus R. (1929). The Suspension Stability of the Blood. Physiol. Rev..

[2] Mateyko G.M., Kopac M.J. (1963). Part II: Isopyknotic Cushioning during High-Speed Centrifugation. Ann. N. Y. Acad. Sci..

[3] Pertoft H., Laurent T.C., Catsimpoolas N. (1977). Isopycnic Separation of Cells and Cell Organelles by Centrifugation in Modified Colloidal Silica Gradients. Methods of Cell Separation.

[4] Pertoft H., Laurent T.C., Låås T., Kågedal L. (1978). Density Gradients Prepared from Colloidal Silica Particles Coated by Polyvinylpyrrolidone (Percoll). Anal. Biochem..

[5] Healthcare G.E. (1985). Cell Separation Media—Methodology and Applications.

[6] Bessis M. (1974). Corpuscles, Atlas of Red Blood Cell Shapes.

[7] Simionato G., Hinkelmann K., Chachanidze R., Bianchi P., Fermo E., van Wijk R., Leonetti M., Wagner C., Kaestner L., Quint S. (2021). Red Blood Cell Phenotyping from 3D Confocal Images Using Artificial Neural Networks. PloS Comput. Biol..

[8] Mohandas N., Evans E. (1994). Mechanical Properties of the Red Cell Membrane in Relation to Molecular Structure and Genetic Defects. Annu. Rev. Biophys. Biomol..

[9] Quint S., Christ A.F., Guckenberger A., Himbert S., Kaestner L., Gekle S., Wagner C. (2017). 3D Tomography of Cells in Micro-Channels. Appl. Phys. Lett..

[10] Kihm A., Quint S., Laschke M.W., Menger M.D., John T., Kaestner L., Wagner C. (2021). Lingering Dynamics in Microvascular Blood Flow. Biophys. J..

[11] Beutler E. (1969). Biochemistry of the Erythrocyte. Experientia.

[12] Agre P., Smith B.L., Hartel-Schenk S. (1990). Biochemistry of the Erythrocyte Rh Polypeptides: A Review. Yale J. Biol. Med..

[13] Wang J., Hertz L., Ruppenthal S., Nemer W.E., Connes P., Goede J.S., Bogdanova A., Birnbaumer L., Kaestner L. (2021). Lysophosphatidic Acid-Activated Calcium Signaling Is Elevated in Red Cells from Sickle Cell Disease Patients. Cells.

[14] Bessis M. (1973). Living Blood Cells and Their Ultrastructure.

[15] Bernhardt I., Ellory J.C. (2003). Red Cell Membrane Transport in Health and Disease.

[16] Kaestner L. (2011). Cation Channels in Erythrocytes—Historical and Future Perspective. Open Biol. J..

[17] Kaestner L. (2015). Channelizing the Red Blood Cell: Molecular Biology Competes with Patch-Clamp. Front. Mol. Biosci..

[18] Thomas S.L.Y., Bouyer G., Cueff A., Egée S., Glogowska E., Ollivaux C. (2011). Ion Channels in Human Red Blood Cell Membrane: Actors or Relics?. Blood Cells Mol. Dis..

[19] Diez-Silva M., Dao M., Han J., Lim C.-T., Suresh S. (2010). Shape and Biomechanical Characteristics of Human Red Blood Cells in Health and Disease. Mrs Bull..

[20] Kaestner L., Bogdanova A., Egee S. (2020). Calcium Channels and Calcium-Regulated Channels in Human Red Blood Cells. Adv. Exp. Med. Biol..

[21] Danielczok J.G., Terriac E., Hertz L., Petkova-Kirova P., Lautenschläger F., Laschke M.W., Kaestner L. (2017). Red Blood Cell Passage of Small Capillaries Is Associated with Transient Ca2+-Mediated Adaptations. Front. Physiol..

[22] Egee S., Kaestner L. (2021). The Transient Receptor Potential Vanilloid Type 2 (TRPV2) Channel—A New Druggable Ca2+ Pathway in Red Cells, Implications for Red Cell Ion Homeostasis. Front. Physiol..

[23] Piccinini G., Minetti G., Balduini C., Brovelli A. (1995). Oxidation State of Glutathione and Membrane Proteins in Human Red Cells of Different Age. Mech. Ageing Dev..

[24] Piomelli S., Seaman C. (1993). Mechanism of Red Blood Cell Aging: Relationship of Cell Density and Cell Age. Am. J. Hematol..

[25] Klein M., Kaestner L., Bogdanova A.Y., Minetti G., Rudloff S., Lundby C., Makhro A., Seiler E., Cromvoirt A., Fenk S. (2021). Absence of Neocytolysis in Humans Returning from a 3-week High-altitude Sojourn. Acta Physiol..

[26] Vettore L., Matteis M.C.D., Zampini P. (1980). A New Density Gradient System for the Separation of Human Red Blood Cells. Am. J. Hematol..

[27] Bosch F.H., Werre J.M., Schipper L., Roerdinkholder-Stoelwinder B., Huls T., Willekens F.L.A., Wichers G., Halie M.R. (2009). Determinants of Red Blood Cell Deformability in Relation to Cell Age. Eur. J. Haematol..

[28] Kaestner L., Minetti G. (2017). The Potential of Erythrocytes as Cellular Aging Models. Cell Death Differ..

[29] Lutz H.U., Stammler P., Fasler S., Ingold M., Fehr J. (1992). Density Separation of Human Red Blood Cells on Self Forming Percoll Gradients: Correlation with Cell Age. Biochim. Biophys. Acta.

[30] Wang J., Wagner-Britz L., Bogdanova A., Ruppenthal S., Wiesen K., Kaiser E., Tian Q., Krause E., Bernhardt I., Lipp P. (2013). Morphologically Homogeneous Red Blood Cells Present a Heterogeneous Response to Hormonal Stimulation. PLoS ONE.

[31] Hertz L., Ruppenthal S., Simionato G., Quint S., Kihm A., Abay A., Petkova-Kirova P., Boehm U., Weissgerber P., Wagner C. (2019). The Evolution of Erythrocytes Becoming Red in Respect to Fluorescence. Front. Physiol..

[32] Mueller T.J., Jackson C.W., Dockter M.E., Morrison M. (1987). Membrane Skeletal Alterations during in Vivo Mouse Red Cell Aging. Increase in the Band 4.1a:4.1b Ratio. J. Clin. Investig..

[33] Inaba M., Gupta K., Kuwabara M., Takahashi T., Benz E.J., Maede Y. (1992). Deamidation of Human Erythrocyte Protein 4.1: Possible Role in Aging. Blood.

[34] Robinson N.E., Robinson A.B. (2004). Molecular Clocks: Deamidation of Asparaginyl and Glutaminyl Residues in Peptides and Proteins.

[35] Vacha J., Agar N.S., Board P.G. (1983). Red Cell Life-Span. Red Blood Cells of Domestic Mammals.

[36] Makhro A., Kaestner L., Bogdanova A. (2017). NMDA Receptor Activity in Circulating Red Blood Cells: Methods of Detection. Methods Mol. Biol. Clifton N. J..

[37] Huisjes R., Makhro A., Llaudet-Planas E., Hertz L., Petkova-Kirova P., Verhagen L.P., Pignatelli S., Rab M.A., Schiffelers R.M., Seiler E. (2019). Density, Heterogeneity and Deformability of Red Cells as Markers of Clinical Severity in Hereditary Spherocytosis. Haematologica.

[38] Makhro A., Huisjes R., Verhagen L.P., del Mañú-Pereira M., Llaudet-Planas E., Petkova-Kirova P., Wang J., Eichler H., Bogdanova A., van Wijk R. (2016). Red Cell Properties after Different Modes of Blood Transportation. Front. Physiol..

[39] Sadafi A., Makhro A., Livshits L., Navab N., Bogdanova A., Albarqouni S., Marr C. (2021). Sickle Cell Disease Severity Prediction from Percoll Gradient Images Using Graph Convolutional Networks. https://arxiv.org/abs/2109.05372v1.

[40] Kaestner L. (2020). Artificial Intelligence Meets Hematology. Transfus. Apher. Sci..

[41] Bogdanova A., Kaestner L. (2020). Early Career Scientists’ Guide to the Red Blood Cell—Don’t Panic!. Front. Physiol..

[42] Abay A., Simionato G., Chachanidze R., Bogdanova A., Hertz L., Bianchi P., van den Akker E., von Lindern M., Leonetti M., Minetti G. (2019). Glutaraldehyde—A Subtle Tool in the Investigation of Healthy and Pathologic Red Blood Cells. Front. Physiol..

[43] Linkert M., Rueden C.T., Allan C., Burel J.-M., Moore W., Patterson A., Loranger B., Moore J., Neves C., Macdonald D. (2010). Metadata Matters: Access to Image Data in the Real World. J. Cell Biol..

[44] McGill R., Tukey J.W., Larsen W.A. (1978). Variations of Box Plots. Am. Stat..

[45] Darras A., Peikert K., Rabe A., Yaya F., Simionato G., John T., Dasanna A.K., Buvalyy S., Geisel J., Hermann A. (2021). Acanthocyte Sedimentation Rate as a Diagnostic Biomarker for Neuroacanthocytosis Syndromes: Experimental Evidence and Physical Justification. Cells.

[46] Darras A., Dasanna A.K., John T., Gompper G., Kaestner L., Fedosov D.A., Wagner C. (2022). Erythrocyte Sedimentation: Collapse of a High-Volume-Fraction Soft-Particle Gel. Phys. Rev. Lett..

[47] Dasanna A.K., Darras A., John T., Gompper G., Kaestner L., Wagner C., Fedosov D.A. (2022). Erythrocyte Sedimentation: Effect of Aggregation Energy on Gel Structure during Collapse. Phys. Rev. E.

[48] Darras A., Breunig H.G., John T., Zhao R., Koch J., Kummerow C., König K., Wagner C., Kaestner L. (2022). Imaging Erythrocyte Sedimentation in Whole Blood. Front. Physiol..

[49] Price C.A. (1982). Centrifugation in Density Gradients.

[50] Flormann D., Aouane O., Kaestner L., Ruloff C., Misbah C., Podgorski T., Wagner C. (2017). The Buckling Instability of Aggregating Red Blood Cells. Sci. Rep..

[51] Lew V.L., Tiffert T. (2013). The Terminal Density Reversal Phenomenon of Aging Human Red Blood Cells. Front. Physiol..

[52] Ermolinskiy P., Lugovtsov A., Yaya F., Lee K., Kaestner L., Wagner C., Priezzhev A. (2020). Effect of Red Blood Cell Aging In Vivo on Their Aggregation Properties In Vitro: Measurements with Laser Tweezers. Appl. Sci..

[53] von Tempelhoff G.-F., Schelkunov O., Demirhan A., Tsikouras P., Rath W., Velten E., Csorba R. (2016). Correlation between Blood Rheological Properties and Red Blood Cell Indices(MCH, MCV, MCHC) in Healthy Women. Clin. Hemorheol. Micro.

[54] Ellory J.C., Hall A.C. (1988). Human Red Cell Volume Regulation in Hypotonic Media. Comp. Biochem. Physiol. A Comp. Physiol..

[55] Sorette M., Shiffer K., Clark M. (1992). Improved Isolation of Normal Human Reticulocytes via Exploitation of Chloride-Dependent Potassium Transport. Blood.

[56] Gottlieb P.A., Sachs F. (2012). Piezo1: Properties of a Cation Selective Mechanical Channel. Channels.

[57] Kaestner L., Egée S. (2018). Commentary: Voltage Gating of Mechanosensitive PIEZO Channels. Front. Physiol..

[58] Bogdanova A., Makhro A., Wang J., Lipp P., Kaestner L. (2013). Calcium in Red Blood Cells-a Perilous Balance. Int. J. Mol. Sci..

[59] Bogdanova A., Kaestner L., Simionato G., Wickrema A., Makhro A. (2020). Heterogeneity of Red Blood Cells: Causes and Consequences. Front. Physiol..

[60] Weems H.B., Lessin L.S. (1984). Erythrocyte Density Distribution in Sickle Cell Anemia. Acta Haematol.-Basel.

[61] Lew V.L., Bookchin R.M. (2005). Ion Transport Pathology in the Mechanism of Sickle Cell Dehydration. Physiol. Rev..

